# Reliability of the Turkish version of the European Obstructive Sleep Apnea Screening (EUROSAS) questionnaire for drivers

**DOI:** 10.1007/s11325-020-02201-2

**Published:** 2020-10-08

**Authors:** Yüksel Peker, Ayse Nilüfer Ozaydin, Ragip Cetinkaya, Ekinsu Kabadayi, Alp Giray Karakucuk, Yeliz Celik, Walter T. McNicholas

**Affiliations:** 1 Department of Pulmonary Medicine, School of Medicine, Koc University, Davutpasa cad, No 4 TR-34010 Zeytinburnu, Istanbul, Turkey; 2 Sahlgrenska Academy, University of Gothenburg, Gothenburg, Sweden; 3 Department of Clinical Sciences, Respiratory Medicine and Allergology, Faculty of Medicine, Lund University, Lund, Sweden; 4 Division of Pulmonary, Allergy, and Critical Care Medicine, School of Medicine, University of Pittsburgh, Pittsburgh, PA USA; 5 Department of Public Health, School of Medicine, Marmara University, Istanbul, Turkey; 6 School Medicine, Marmara University, Istanbul, Turkey; 7 Department of Respiratory and Sleep Medicine, St. Vincent’s Hospital Group, School of Medicine, University College, Dublin, Ireland

**Keywords:** Obstructive sleep apnea, Traffic safety, Daytime sleepiness, Questionnaire

## Abstract

**Purpose:**

The European Union Driver License Committee recently developed a questionnaire as a screening tool for obstructive sleep apnea (OSA) named the European Obstructive Sleep Apnea Screening (EUROSAS) questionnaire for drivers. We sought to address the reliability of the Turkish version of this questionnaire.

**Methods:**

The EUROSAS was translated into Turkish. Using a “test-retest approach”, data were collected twice with a 15-day interval among 150 participants (50 professional male drivers [PMD], 50 non-professional male drivers [NPMD], and 50 non-professional female drivers [NPFD]). The EUROSAS score ranges between 2 and 25, with scores ≥ 10 suggesting the presence of OSA.

**Results:**

The median EUROSAS scores in the first test were 8.0 (interquartile range [IQR] 6.8–12.0) in PMD, 8.0 (IQR 6.0–11.0) in NPMD, and 5.0 (IQR 4.0–8.0) in NPFD (*p* < 0.001). Corresponding values in the retest were 9.5 (IQR 7.0–13.0), 8.0 (IQR 6.0–10.0), and 5.0 (IQR 4.0–8.0), respectively (*p* < 0.001). The EUROSAS score ≥ 10 was found among 34% in the first test and 50% in the retest in PMD (ns), 34% vs 24% in NPMD (ns), and 8% vs 16% in NPFD (ns). There was a positive correlation between the tests (*r* = 0.864, *p* < 0.001), and Cronbach’s alpha value for the whole group was 0.477 (0.514 for PMD, 0.512 for NPMD, and 0.543 NPFD, respectively).

**Conclusions:**

The EUROSAS–Turkish version is easy to understand and is reproducible. However, the test-retest reliability level is poor among the Turkish drivers. Further validation of the questionnaire by objective sleep studies and fitness-to-drive testing is necessary.

**Electronic supplementary material:**

The online version of this article (10.1007/s11325-020-02201-2) contains supplementary material, which is available to authorized users.

## Introduction

Motor vehicle accidents (MVA) are one of the most important causes of death and injury in Turkey [1] and worldwide [2, 3]. According to accident statistics in Turkey, 95% of MVAs are caused by drivers, mainly due to high speed, alcohol, substance use, and sleepiness [1]. The most common medical disorder causing sleepiness is obstructive sleep apnea (OSA). According to a questionnaire-based study, the Turkish Adult Population Epidemiology of Sleep (TAPES) [4], including a nationwide representative sample of 5021 adults, the OSA prevalence in Turkey is around 14%, based on the Berlin questionnaire [5], and excessive daytime sleepiness (EDS) is 5.4% based on the Epworth Sleepiness Scale (ESS) scores [6, 7].

During the last decades, several attempts have been intensively debated regarding the development of national and international strategies to screen high-risk drivers for sleepiness and OSA. The increasing awareness of OSA as a risk factor for MVAs, which is reversed by effective treatment with continuous positive airway pressure (CPAP), has led to a revision of annex III of the European Union (EU) directive on driving licenses that is subject to mandatory implementation by all member states from December 31, 2015 [8]. This directive was based on the recommendations from a working group established by the Transport and Mobility Directorate of the European Commission in 2012 [9]. Accordingly, Turkish legislation has also been revised for regulation of driving license issues for individuals with OSA. For instance, a full-night polysomnography (PSG) is recommended and treatment should be initiated as soon as possible when the OSA diagnosis is confirmed [10]. However, given the high prevalence of OSA in general population and the limited resources for PSG, there are many controversies and difficulties in implementation of these rules in practice.

In this context, we aimed to use a questionnaire that was developed by the European Union Driver License Committee as a screening tool for OSA (Supplement 1) [9]. We named the questionnaire the European Obstructive Sleep Apnea Screening (EUROSAS) questionnaire for drivers and sought to test the reliability of its Turkish version.

## Methods

### Participants

This methodological study was conducted at the Marmara University School of Medicine, Istanbul in 2017. The study participants comprised 150 volunteers, of whom 50 were professional male drivers (PMD), 50 non-professional male drivers (NPMD), and 50 non-professional female drivers (NPFD). All individuals had driving licenses for at least 3 years, and had been driving actively twice a day. The PMDs were recruited from five different local taxi stations (Kayışdağı Merkez, Fındıklı Merkez, Ataşehir Site, Türkiş Blokları, and Fındıklı) in the Asian site of Istanbul. The PMDs were working two shifts from 3 a.m. to 3 p.m. for 1 week, and from 3 p.m. to 3 a.m. the other week, respectively. The non-professional drivers were recruited from students and staff of the Medical School as well as from local workers of the market in the Başıbüyük region, where the Medical School is located. The students were preclinical and had no night duties, and none of the other non-professional drivers was working shifts.

### Procedure and measures

The EUROSAS questionnaire (Appendix 1) was translated from English to Turkish by YP (Version 1). The Turkish version was translated back to English, and then to Turkish again by two independent translators, respectively. Each participant completed the EUROSAS questionnaire twice during the daytime with a 15-day time interval (see Appendix 2 for the Turkish versions). The EUROSAS questionnaire consists of 11 items in total. The first four items refer to demographic characteristics, including gender, age, weight, and height, and the following six items refer to history of sleepiness while driving, a serious MVA due to EDS, history of snoring, witnessed apneas and non-restorative sleep, and prevalent hypertension [8]. The last item refers to total score of the ESS, which is the most widely used questionnaire at sleep clinics worldwide for the purpose of assessing the presence of daytime sleepiness [6, 7]. All EUROSAS questions are attributed a value, reflecting the strength of the relationship between a given answer and the risk for MVAs or the probability of suffering from OSA, based on a consensus minimal agreement among the members of the working group [9]. The possible value for the EUROSAS ranges from 2 to 24, and individuals with 10 points or above on the EUROSAS scale were considered to be positive for OSA [9].

### Statistical analysis

Collected data were analyzed through IBM Statistical Package for the Social Sciences (SPSS), version 25.0 for Windows® system (SPSS® Inc., Chicago, Illinois, USA). Descriptive statistics were given as means ± SD and categorical variables as numbers (percentages). Socio-demographic characteristics were analyzed by using chi-square test across three groups. Assumption of normality was tested using the Shapiro-Wilk test. The EUROSAS scores were given as median and interquartile range (IQR) values. The comparison of the groups was tested by Kruskal-Wallis test. For post hoc pairwise comparisons, Mann Whitney *U* test with Bonferroni correction was performed. The internal consistency of the EUROSAS was measured with Cronbach’s alpha value, which estimates the correlations between 11 items. Test-retest reliability was evaluated with respect to the bivariate correlation between the first and the second tests. All statistical tests were two-sided, and *p* < 0.05 was considered statistically significant.

## Results

As shown in Table 1, most of the participants were older than 30 years, non-obese, and were married. Few had comorbidities. Almost half of the entire study population were university graduates with the highest proportion (90%) among the NPFDs, and the lowest (10.0%) among the PMDs (*p* < 0.001).

**Table 1:** Socio-demographic characteristics of the participants in the study

Socio-demographic characteristics	All participants (*n* = 150)		Professional male drivers (*n* = 50)		Non-professional male drivers (*n* = 50)		Non-professional female drivers (*n* = 50)		*P*	
	*n*	%	*n*	%	*n*	%	*n*	%		
Age > 30 years	129	86.0	43	86.0	39	78.0	47	94.0	0.07	
Education										
Pri/sec. school	41	27.3	29	58.0	11	22.0	1	2.0		
High school	37	24.7	16	32.0	17	34.0	4	8.0	< 0.001	
University	72	48.0	5	10.0	22	44.0	45	90.0		
Marital Status										
Currently married	115	76.7	39	78.0	40	80.0	36	72.0		
Divorced	15	10.0	5	10.0	2	4.0	8	16.0	0.166	
Never married	20	13.3	6	12.0	8	16.0	6	12.0		
BMI										
≤ 30	124	82.7	39	78.0	41	82.0	44	88.0		
31–35	17	11.3	7	14.0	5	10.0	5	10.0	0.596	
≥ 36	9	6.0	4	8.0	4	8.0	1	2.0		
Disease										
Hypertension	15	10.0	9	18.0	2	4.0	4	8.0	0.056	
Diabetes mellitus	9	6.0	5	10.0	2	4.0	2	4.0	0.345	
Cardiovascular	4	2.7	3	6.0	0	0.0	1	2.0	0.166	
Respiratory	8	5.3	2	4.0	0	0.0	6	12.0	0.025	

**Fig. 1:**
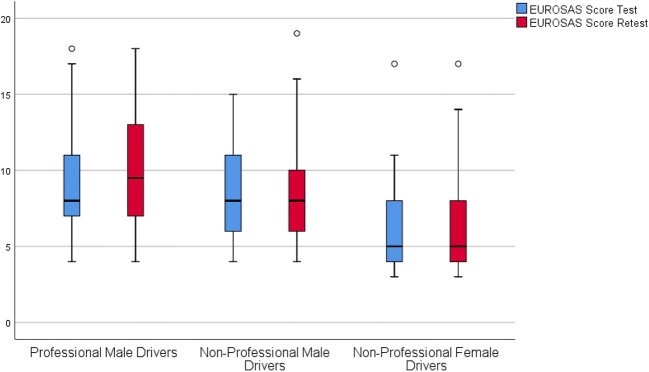
Median values of the EUROSAS test and retest scores of the study participants

The normality assumption of the total EUROSAS scores was violated (*W* = 0.94, *p* < 0.001 for the first test, and *W* = 0.92, *p* < 0.001 for the retest, respectively). As shown in Fig. 1, the female drivers had significantly lower median scores than the male drivers; in the first test 8.0 (IQR 6.8–12.0) in PMD, 8.0 (IQR 6.0–11.0) in NPMD, and 5.0 (IQR 4.0–8.0) in NPFD (*p* < 0.001). Corresponding values in the retest were 9.5 (IQR 7.0–13.0), 8.0 (IQR 6.0–10.0), and 5.0 (IQR 4.0–8.0), respectively (*p* < 0.001).

**Fig. 2:**
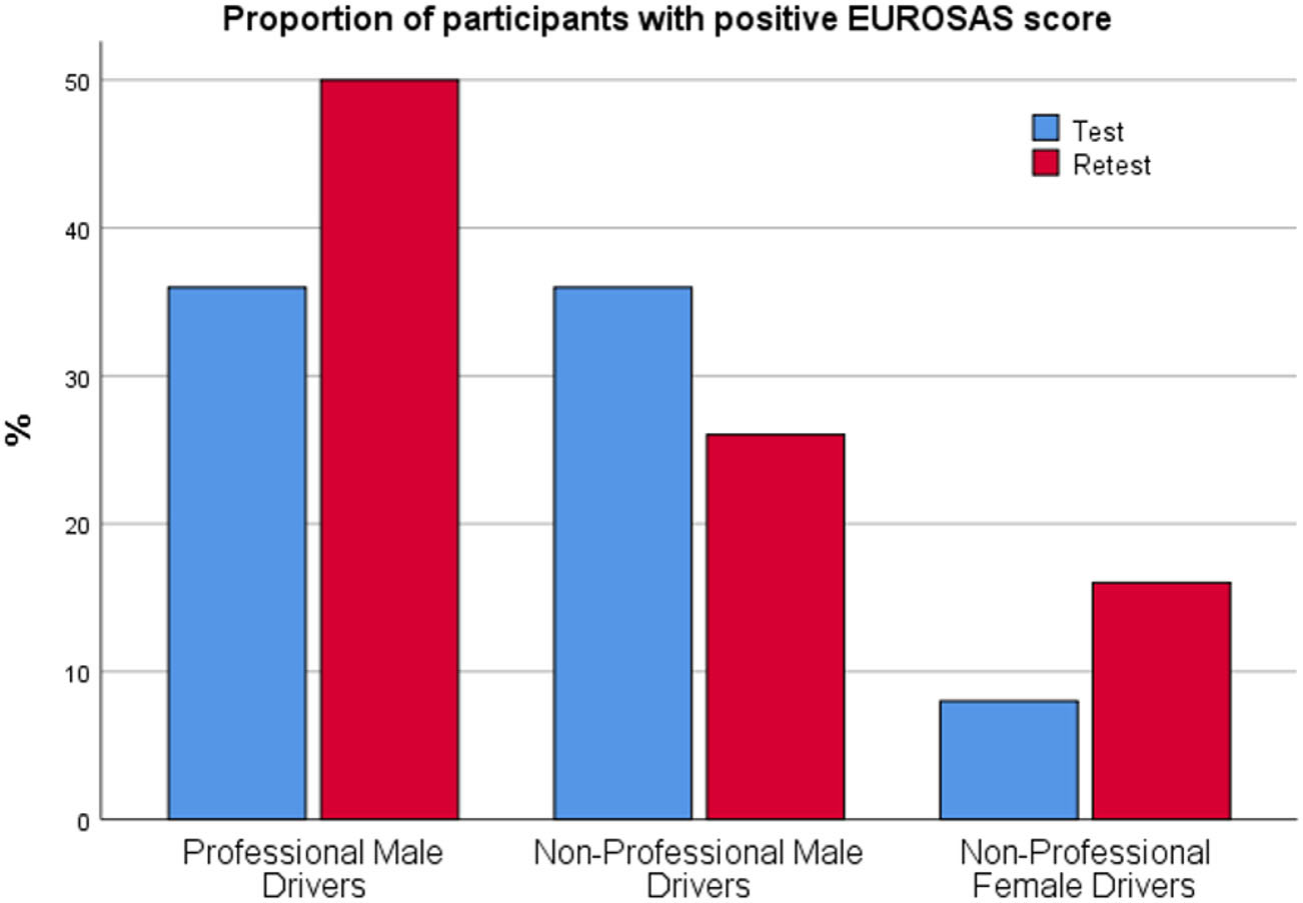
Proportion of participants with positıve EUROSAS scores

**Fig. 3:**
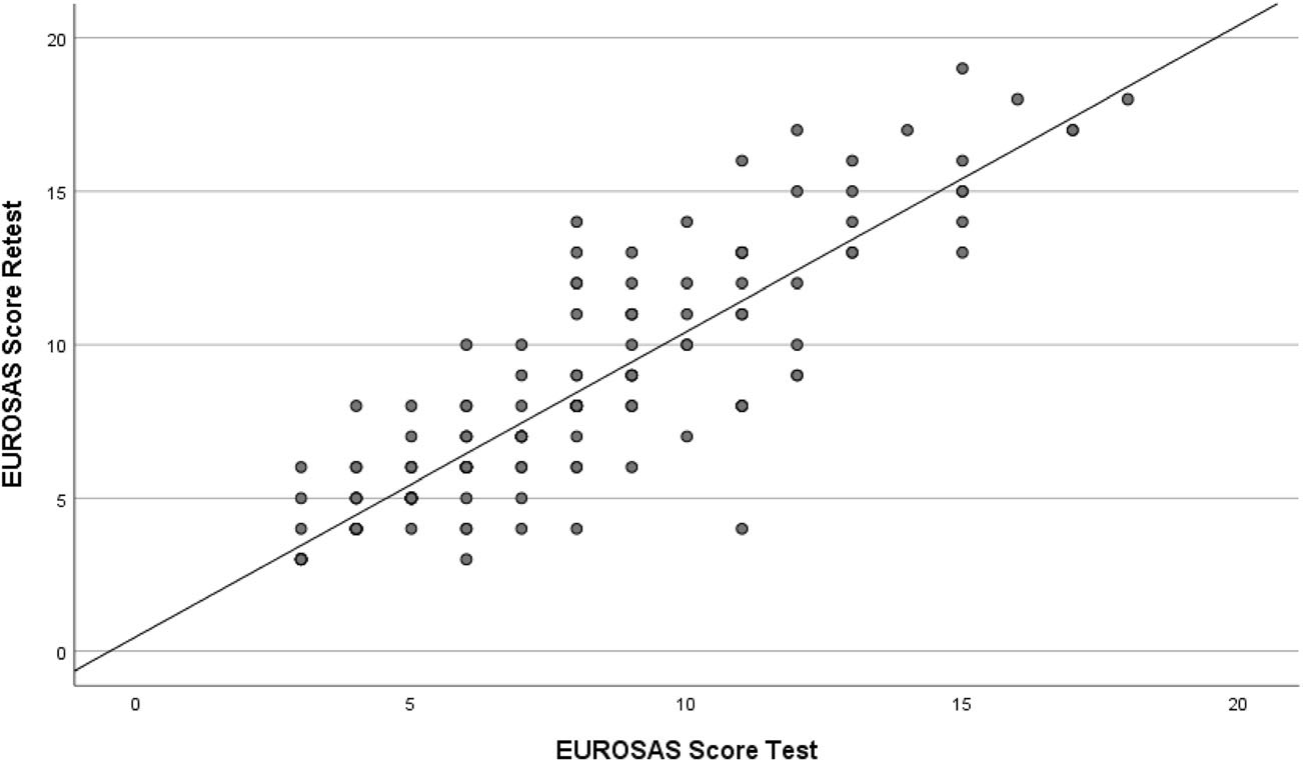
Correlation between EUROSAS test and retest scores in the study population

As demonstrated in Fig. 2, the proportion of participants with EUROSAS score ≥ 10 increased from 34% in the first test to 50% in the retest among the PMD (ns). The corresponding values were 34% vs 24% in NPMD (ns), and 8% vs 16% in NPFD (ns). There was a strong positive correlation between two measurements (*r* = 0.864; *p <* 0.001), indicating a good reproducibility of the EUROSAS scores (Fig. 3). Cronbach’s alpha coefficients of the EUROSAS for the entire population were calculated as 0.463 for the first test, and 0.562 for the retest.

As presented in Table 2, range of mean item scores was relatively wide on both measurements. The highest mean score was yielded on the item 1 (gender), which was calculated as 1.67. The lowest mean score was yielded on the item 8 (Have you been told that your breathing stops during sleep?) which was 0.09 for the first test and 0.11 for the retest. Likewise, the range of the item total correlation coefficients was also considerably broad (Table 2). The lowest item total correlation was yielded on the item 10 (Do you suffer arterial hypertension?), which was 0.031 for the first test, and 0.048 for the retest, respectively. The item 11 (ESS) showed the highest item total correlation, 0.346 on the first test, and 0.491 on the retest, respectively (Table 2).

**Table 2:** Item analysis

EUROSAS	Mean + SD		Item to total correlation		Cronbach’s alpha if item is deleted	
	Test	Retest	Test	Retest	Test	Retest
Item 1	1.67 ± 0.47	1.67 ± 0.47	0.25	0.220	0.430	0.547
Item 2	1.14 ± 0.34	1.14 ± 0.34	− 0.117	− 0.124	0.489	0.585
Item 3 and 4	1.23 ± 0.54	1.23 ± 0.54	0.197	0.257	0.439	0.539
Item 5	1.11 ± 1.44	0.97 ± 1.40	0.326	0.450	0.374	0.459
Item 6	0.11 ± 0.64	0.16 ± 0.78	0.126	0.205	0.455	0.546
Item 7	0.75 ± 0.92	0.76 ± 0.92	0.344	0.442	0.375	0.478
Item 8	0.09 ± 0.28	0.11 ± 0.31	0.133	0.289	0.458	0.547
Item 9	0.96 ± 0.96	1.15 ± 0.96	0.139	0.138	0.457	0.568
Item 10	0.16 ± 0.53	0.22 ± 0.60	0.031	0.048	0.474	0.574
Item 11	0.65 ± 0.28	0.89 ± 1.45	0.346	0.491	0.360	0.438

## Discussion

The main conclusion of the current study is that the EUROSAS questionnaire is easy to understand and is applicable with good reproducibility among Turkish professional and non-professional drivers. However, the test-retest reliability level seems to be poor.

To our knowledge, this is the first reliability study of a screening questionnaire developed by the EU Driver License Committee, and the first definition of the acronym EUROSAS in Europe. The increasing consciousness about OSA as a risk factor for MVAs [2, 3], and the observed beneficial effect of CPAP treatment in reducing this risk in retrospective studies [11] has led to a revision of annex III of the EU directive on driving licenses that is a mandatory document for implementation by all member states from December 31, 2015 [8, 9]. As summarized in an editorial [12], this directive includes: “(a) applicants or drivers in whom a moderate or severe OSA syndrome is suspected shall be referred for further authorized medical advice before a driving license is issued or renewed. They may be advised not to drive until confirmation of the diagnosis; (b) driving licenses may be issued to applicants or drivers with moderate or severe OSA syndrome who show adequate control of their condition and compliance with appropriate treatment and improvement of sleepiness, if any, confirmed by authorized medical opinion; (c) applicants or drivers with moderate or severe OSA syndrome under treatment shall be subject to a periodic medical review, at intervals not exceeding 3 years for noncommercial drivers, and 1 year for commercial drivers, with a view to establish the level of compliance with the treatment, the need for continuing the treatment and continued good vigilance.” [12].

In accordance with the aforementioned EU directive [8, 9], the Turkish legislation has been revised as following [10]: (a) subjects with severe OSA (AHI > 30/h), and subjects with moderate OSA (AHI 15–30/h) with documented daytime sleepiness should not be given driving license unless this condition is treated; (b) the efficient treatment should be documented to include the severity of OSA at baseline, response to treatment, compliance with CPAP; (c) the applicants with BMI > 33 kg/m^2^ should undergo full-night PSG regardless of symptoms; (d) the applicants with witnessed apneas and daytime sleepiness should undergo full-night PSG regardless of BMI. Given that access to OSA diagnosis and treatment is already limited for symptomatic patients, the current legislation has led to an even higher number of requests for specialist evaluation for drivers with BMI >33kg/m^2^ regardless of symptoms, and lengthened the waiting lists for PSG. Thus, screening for OSA by using a full-night PSG on a large scale is not feasible in Turkey. On the other hand, though OSA increases the risk of traffic accidents, the disorder is associated with EDS in only one-fifth of the patients according to populationstudies [13]. The majority of evidence support that driving risk in OSA is closely related to the degree of daytime sleepiness rather than the severity of OSA in terms of AHI [14, 15]. There is yet no consensus on the definition of “a high-risk driver” other than a report from the American Thoracic Society [16]. The report suggests that a high-risk driver is the one who has moderate to severe daytime sleepiness, and a recent unintended MVA or a near-miss attributable to the sleepiness, fatigue, orinattention [16].

In the medical literature, there have been several screening questionnaires for OSA in general populations and sleep clinic cohorts [5, 6]. The current EUROSAS questionnaire for drivers may be a good preliminary screening tool to identify the high-risk drivers, especially in countries that have less access to full polysomnography for diagnosing OSA, and consequently initiating CPAP treatment. As expected, the probability of OSA was less common among the female drivers. In males, the risk of having OSA showed an increasing pattern among the professional drivers while there was a decreasing trend among the non-professionals drivers. Though this might be explained by the regression toward the mean, it might also reflect the initial legal concerns of the professional drivers. Nevertheless, there was a strong correlation between the first and second measurements in the entire cohort, suggesting a good reproducibility of the EUROSAS questionnaire. Of note, the threshold of 10 is arbitrary, and requires further validation.

As reflected by the Cronbach’s alpha coefficients, the reliability of the EUROSAS questionnaire was poor in the current study population. Expectedly, the highest mean score was found in item 1 (gender), and the lowest mean score was found in item 8 (Have you been told your breathing stops during your sleep?) while the item 11 (ESS) showed the highest item total correlation (Table 2). The lowest item total correlation was found in item 10 (Do you suffer arterial hypertension?), which might be related to the self-reported hypertension diagnosis, and may not be sufficiently reliable for evaluation of the EUROSAS scores. Thus, given the broad range of the item scores both in the first test and the retest, our results should be interpreted cautiously, and regarded as a pilot evaluation of the EUROSAS questionnaire for drivers.

### Study limitations

Our study has several limitations. First, subjective EDS in OSA patients is open for bias by a driver who seeks to underestimate OSA severity for legal issues. Second, there is no objective sleep study in the current report, so the EUROSAS questionnaire has yet not been validated against a full-night polysomnography for OSA diagnosis and against a maintenance of wakefulness test or a real simulator-based fitness-to-drive test. Third, the EUROSAS questionnaire does not consider providing data regarding shift work and the work load of the specific test day. These aspects need to be considered in the future evaluations and validations of the questionnaire for drivers.

## Conclusions

We conclude that the EUROSAS–Turkish version is easy to understand and is reproducible among the drivers in Turkey. However, the test-retest reliability level is poor, and the probability of having OSA seems to be underestimated at the first test among the PMDs. This underestimation may reflect the drivers’ concerns for legal issues for losing their driving licenses. Further validation of the questionnaire by objective sleep studies and fitness-to-drive testing is required.

## Electronic supplementary material

ESM 1 (PDF 205 kb)

ESM 2 (PDF 382 kb)
