# Supplementary material for: Reliability of the Turkish version of the European Obstructive Sleep Apnea Screening (EUROSAS) questionnaire for drivers
Source: Sleep Breath. 2020 Oct 8;25(2):907–13. doi: 10.1007/s11325-020-02201-2 (PMC8195917; doi:10.1007/s11325-020-02201-2)
Supplement: Supplementary file 1 — (PDF 205 kb) [file 11325_2020_2201_MOESM1_ESM.pdf]

Proposed questionnaire to screen for OSAS

1. Gender
2. Age
3. Weight
4. Height
5. Did it already happen to you to doze off while driving? YES NO DON'T KNOW
6. Did you have a serious accident (with personal injuries or property damage) due to sleepiness in the last 3 years? YES NO DON'T KNOW
7. Do you usually snore loudly almost every night? YES NO DON'T KNOW
8. Have you been told your breathing stops during your sleep? YES NO DON'T KNOW
9. Do you usually wake up refreshed after a full night sleep? YES NO DON'T KNOW
10. Do you suffer from, or are you being treated for, Arterial Hypertension? YES NO DON'T KNOW
11. Please complete the questionnaire on usual daytime sleepiness, called the Epworth Sleepiness Scale, on the next page

The questions are attributed a value, reflecting the strength of the association between a given answer and the risk of MVAs or the possibility of suffering from OSAS, as well as the level of uncertainty concerning this strength. This is based on a consensus minimal agreement among the members of the working group.

Q. 1: Female =1; Male =2

Q. 2: Age below 30 yo= 2; Age 31 yo or above= 1

Q. 3-4: A BMI below 30 kg/m^2= 1, 31-35 kg/m^2= 2; 36 kg/m^2 or higher= 3

Q. 5: A positive answer= 3; negative answer= 0; don't know= 2

Q. 6: A positive answer= 4; negative answer= 0; don't know= 3

Q. 7: A positive answer= 2; negative answer= 0; don't know= 1

Q. 8: A positive answer= 1; negative answer= 0; don't know= 0

Q. 9: A negative answer= 2; positive answer= 0; don't know 1

Q. 10: A positive answer = 2; negative answer = 0; don't know = 1

ESS: From 11 to 14= 2; 15 or higher: 4

The maximal possible value for this *sui generis* questionnaire is 24. A male middle-aged obese male has already a value of 5 or 6 (depending on the level of obesity) from the start. If he has 3 characteristics linked to OSAS (questions 7, 8, 9 and 10), he will score 10 points. If he has previous MVAs or probable sleep episodes at the wheel, he will score 8 or 9 points, and if he is mildly sleepy or has some evidence of sleep apnoea syndrome, he will also reach the 10 points value.
