# Supplementary material for: Reliability of the Turkish version of the European Obstructive Sleep Apnea Screening (EUROSAS) questionnaire for drivers
Source: Sleep Breath. 2020 Oct 8;25(2):907–13. doi: 10.1007/s11325-020-02201-2 (PMC8195917; doi:10.1007/s11325-020-02201-2)
Supplement: Supplementary file 2 — (PDF 382 kb) [file 11325_2020_2201_MOESM2_ESM.pdf]

The Turkish Version of the EUROSAS Questionnaire

First Test

1) Cinsiyet: 1. K 2. E
2) Doğum yılı: …………
3) Boyunuz: .... cm
4) Kilonuz: ....kg

5) Siz hiç araba kullanırken uyuklar gibi oldunuz mu?	EVET	HAYIR	BİLMİYORUM
6) Son 3 yıl içinde, uyuklama hali nedeniyle, taşıt kullanırken, kişi ya da araç hasarına neden olan herhangi bir trafik kazası geçirdiniz mi?	EVET	HAYIR	BİLMİYORUM
7) Hemen her gece yüksek sesle horlar mısınız?	EVET	HAYIR	BİLMİYORUM
8) Uykudayken nefesinizin durduğunu söylüyorlar mı?	EVET	HAYIR	BİLMİYORUM
9) Genellikle, gece uyuduktan sonra sabah uyandığınızda kendinizi dinç/zinde/dinlenmiş hisseder misiniz?	EVET	HAYIR	BİLMİYORUM
10) Yüksek tansiyonunuz olduğu söylendi mi?	EVET	HAYIR	BİLMİYORUM

Aşağıdaki durumlarda sadece yorgun hissediyor olmaya göre, uyuklamaya ya da uykuya dalmaya ne kadar eğilimlisiniz?

0 = Hiç
1 = Hafif derecede
2 = Orta derecede
3 = İleri derecede

**Durum**	**Uyuklama İhtimali (0-3)**
Otururken ve okurken	
Televizyon seyrederken	
Umumi bir yerde (örn: sinema, tiyatro ya da bir toplantıda) bir şey yapmadan otururken	
Bir arabada bir saat boyunca ara vermeden yolcu olarak bulunurken	
Öğleden sonra koşullar izin verdiğinde dinlenmek için uzanırken	
Biriyle oturup konuşurken	
(Alkol almadığınız) Bir öğle yemeği sonrası sessizce otururken	
Bir arabada, trafikte birkaç dakika duruyorken	

The Turkish Version of the EUROSAS Questionnaire

Retest

1) Cinsiyet: 1. K 2. E
2) Doğum yılı: …………......
3) Boyunuz: ....cm
4) Kilonuz: ....kg

5) Hiç araba kullanırken uyukladığınız oldu mu?	EVET	HAYIR	BİLMİYORUM
6) Geçen 3 yıl içerisinde, araba kullanırken uyukladığınız için kaza yapıp kişilere veya araçlara zarar verdiniz mi?	EVET	HAYIR	BİLMİYORUM
7) Hemen her gece horlar mısınız?	EVET	HAYIR	BİLMİYORUM
8) Size uykuda nefesinizin durduğunu söyleyen oldu mu?	EVET	HAYIR	BİLMİYORUM
9) Sabahları genellikle dinlenmiş ve canlı olarak mı kalkarsınız?	EVET	HAYIR	BİLMİYORUM
10) Yüksek tansiyonunuz var mı?	EVET	HAYIR	BİLMİYORUM

Aşağıda belirtilen durumlarda uyuklama veya uyuyakalma durumunuz nedir? Yanıt verirken size en uygun seçeneği belirleyiniz:

0 = Hiç
1 = Biraz
2 = Orta derecede
3 = Çok

**Durum**	**Uyuklama/uyuyakalma (0-3)**
Otururken ve okurken	
TV izlerken	
Sinema, tiyatro gibi bir yerde otururken	
1 saatlik bir araba yolculuğunda, yolcu olarak seyahat ederken	
Öğlen yemeğinden sonra uygun ortam varsa, uzanmış dinlenirken	
Birisiyle konuşurken	
Alkolsüz bir öğlen yemeğinden sonra sessizce otururken	
Arabada, trafikte beklerken	
